# The novel high-pressure/high-temperature compound Co_12_P_7_ determined from synchrotron data

**DOI:** 10.1107/S2056989020012657

**Published:** 2020-09-22

**Authors:** Claire Zurkowski, Barbara Lavina, Stella Chariton, Sergey Tkachev, Vitali Prakapenka, Andrew Campbell

**Affiliations:** a University of Chicago, Department of the Geophysical Sciences, 5734 S. Ellis Ave, Chicago IL, 60637, USA; b X-ray Science Division, Advanced Photon Source, Argonne National Laboratory, Argonne, IL 60439, USA; c University of Chicago, GeoSoilEnviro Center for Advanced Radiation Sources, Chicago, IL 60637, USA

**Keywords:** crystal structure, synchrotron, high-pressure synthesis, cobalt phosphide, *M*_12_P_7_ phase

## Abstract

The structural properties of cobalt phosphides were investigated at high pressures and temperatures to better understand the behavior of metal-rich phosphides in Earth and planetary inter­iors. Using single-crystal X-ray diffraction synchrotron data and a laser-heated diamond anvil cell, we discovered a new high pressure–temperature (*HP–HT*) cobalt phosphide, Co_12_P_7_, dodeca­cobalt hepta­phosphide, synthesized at 27 GPa and 1740 K, and at 48 GPa and 1790 K. Co_12_P_7_ adopts a structure initially proposed for Cr_12_P_7_ (space-group type *P* , *Z* =1), consisting of chains of edge-sharing CoP_5_ square pyramids and chains of corner-sharing CoP_4_ tetra­hedra. This arrangement leaves space for trigonal–prismatic channels running parallel to the *c* axis. Coupled disordering of metal and phospho­rus atoms has been observed in this structure for related *M*_12_P_7_ (*M* = Cr, V) compounds, but all Co and P sites are ordered in Co_12_P_7_. All atomic sites in this crystal structure are situated on special positions. Upon decompression to ambient conditions, peak broadening and loss of reflections at high angles was observed, suggesting phase instability.

## Abstract 2

Co_12_P_7_, synthesized at high pressure/temperature conditions, crystallizes isotypically with ordered Cr_12_P_7_ in space-group type *P* 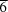.

## Chemical context   

Cobalt phosphides have previously been examined in the context of binary phase relations and thermodynamics (Okamoto & Massalski, 1990 ▸; Schlesinger, 2002 ▸) and have gained attention for their unique conductive properties (Prins & Bussell, 2012 ▸; Popczun *et al.*, 2014 ▸; Pan *et al.*, 2016 ▸; Pramanik *et al.*, 2017 ▸), magnetic properties (Fujii *et al.*, 1988 ▸; Jeitschko *et al.*, 1978 ▸; Jeitschko & Jaberg, 1980 ▸; Reehuis & Jeitschko, 1989 ▸), and ability to store lanthanide cations (Jeitschko *et al.*, 1978 ▸). Cobalt phosphides also serve as structural analogs to iron-rich phosphides and sulfides in planetary core-forming alloys. Previous studies of CoP and Co_2_P indicate that their phase relations tend to precede in pressure the stability of isostructural Fe-phosphides and Fe-sulfides (Rundqvist, 1960 ▸; Ellner & Mittemeijer, 2001 ▸; Dera *et al.*, 2008 ▸; Tateno *et al.*, 2019 ▸; Rundqvist, 1962 ▸; Ono & Kikegawa, 2006 ▸; Ono *et al.* 2008 ▸). Hence, understanding the behavior of cobalt phosphides at high pressures provides insight into the ultra-high pressure behavior of iron sulfides and phosphides.

There are few structures reported in the literature for transition-metal phosphides with the composition *M*_12_P_7_. Baurecht *et al.* (1971 ▸) first examined Cr_12_P_7_ and determined that it adopts a hexa­gonal lattice with space group *P* 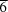, *Z* = 1. The structure consists of columns of alternating tetra­hedral and pyramidal polyhedra and columns of stacked triangular–prismatic polyhedra extending along the *c-*axis direction. Chromium atoms occupy half of all possible tetra­hedral and pyramidal sites while the triangular–prismatic sites are empty (Baurecht *et al.*, 1971 ▸). The polyhedra in the unit cell can be described as Cr_9_^P^Cr_3_^T^[] _2_^Pr^P_7_ (P = pyramidal, T = tetra­hedral, Pr = trigonal–prismatic, [] = empty site) (Maaref *et al.*, 1981 ▸). Coupled disordering of two half-atoms of the corresponding metal with two half-atoms of phospho­rus within the tetra­hedral and pyramidal sites has been observed in this structure for compounds Th_7_S_12_, V_12_P_7_, and Cr_12_P_7_, increasing the symmetry to the *P*6_3_/*m* space group (Zachariasen, 1949 ▸; Olofsson & Ganglberger 1970 ▸; Chun & Carpenter, 1979 ▸).

At ambient conditions the *M*_12_P_7_ composition is not observed in the binary systems with *M* = Co, Ni, Fe. Dhahri (1996 ▸) concluded that Co_12_P_7_, Ni_12_P_7_ and Fe_12_P_7_ do not occur in the Cr_12_P_7_ structure type at ambient conditions because, unlike Cr and V, the elements Co, Ni and Fe do not preferentially occupy pyramidal sites. In support of this conclusion, the Zn_2_Fe_12_P_7_ structure type (*P* 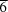, *Z* = 1) with many structural similarities to the Cr_12_P_7_ structure type, has been observed in *Ln*_2_ *M*_12_P_7_ (*Ln* = rare-earth element; *M* = Co, Ni, Fe) compounds where the pyramidal-to-tetra­hedral site ratio is 1:3 (Jeitschko *et al.*, 1978 ▸; Jeitschko & Jaberg, 1980 ▸; Reehuis & Jeitschko, 1989 ▸). Ordering is present in the Co-, Fe-, Ni-rich Zn_2_Fe_12_P_7_ isomorphs (Jeitschko *et al.*, 1984 ▸). No other structure types for the composition *M*_12_P_7_ (*M* = Co, Ni, Fe) have been reported so far.

The effect of pressure and temperature on stabilizing Co in both the tetra­hedral and pyramidal sites and ordering of Co and P in the Cr_12_P_7_-type structure has not been examined previously. In the current study, we report the synthesis of a Co_12_P_7_ phase at 27 GPa and 1750 K, and at 48 GPa and 1790 K; both phases are isostructural and crystallize in space group *P* 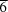. Structure refinements revealed that Co and P sites are ordered in the high *P–T* structure and Co atoms occupy tetra­hedral and pyramidal coordinations. Using single-crystal diffraction techniques, we report refined atomic coordinate sites of Co_12_P_7_ at 48 GPa and 15 GPa.

## Structural commentary   

**Figure 1:**
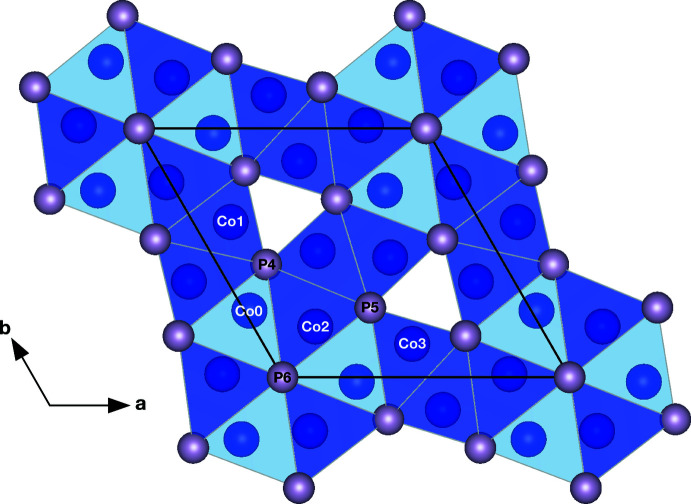
Crystal structure of Co_12_P_7_ based on the 48 GPa data set with atoms of the asymmetric unit labeled. CoP_4_ tetra­hedra are shaded in cyan and CoP_5_ square pyramids are shaded in violet.

Refinement of the structure confirms that Co_12_P_7_ assumes the ordered Cr_12_P_7_ structure type (Baurecht *et al.*, 1971 ▸; Chun & Carpenter, 1979 ▸). Two of the Co sites (Co0, Co1) occupy Wyckoff position 3 *j* (point group symmetry *m*..), the other two Co sites (Co2, Co3) Wyckoff position 3 *k* (*m*..), one P site (P5) Wyckoff position 3 *j*, one P site (P4) Wyckoff position 3 *k*, and one P site (P6) Wyckoff position 1 *a* (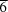..). The Co sites occupy tetra­hedral (cyan) and pyramidal (violet) sites as imaged in Fig. 1 ▸. Chains of edge-sharing CoP_5_ square pyramids and chains of corner-sharing CoP_4_ tetra­hedra build up the framework with trigonal–prismatic channels running parallel to the *c* axis.

**Figure 2:**
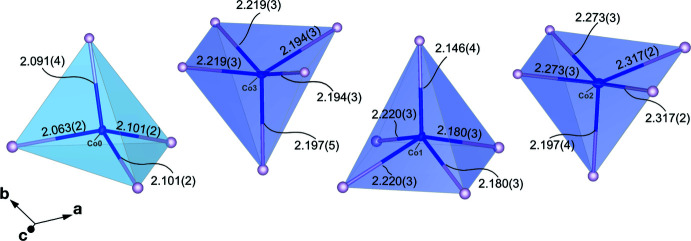
Co—P polyhedra as observed in the Co_12_P_7_ structure (48 GPa data set) showing varying degrees of volume and distortion, qu­anti­fied in Table 1 ▸. CoP_4_ tetra­hedra are shaded in cyan and CoP_5_ square pyramids are shaded in violet. Displacement ellipsoids are drawn at the 50% probability level.

Ranges of inter­atomic Co—P distances and polyhedral volumes are provided in Table 1 ▸ and Fig. 2 ▸ with CoP_4_ tetra­hedra represented by a cyan polyhedron and CoP_5_ pyramids represented by violet polyhedra. Co0 atoms occupy a distorted tetra­hedral site with one P atom at a short distance, two at inter­mediate distances and one at a long distance (Table 1 ▸, Fig. 2 ▸). Co1 and Co2 atoms occupy square pyramids with two inter­mediate and two long inter­atomic distances at the base. Co3 atoms occupy a less distorted square pyramid with two elongated and two truncated bonds at the base (Fig. 2 ▸). Inter­atomic distances at 48 GPa range from 2.063 (2)–2.102 (2) Å in the tetra­hedral polyhedra, 2.147 (4)–2.220 (4) Å for Co1—P polyhedra, 2.197 (4)–2.317 (2) Å for Co2—P polyhedra and 2.194 (3)–2.219 (3) Å for Co3—P polyhedra (Table 1 ▸). These inter­atomic distances are comparable to those observed in Co_2_P and CoP (Rundqvist 1960 ▸, 1962 ▸).

A grain of Co_12_P_7_ was decompressed to ambient conditions where 44 total reflections were identified in reciprocal space and indexed to a unit cell of *a* = 8.47 (1) Å, *c* = 3.37 (1) Å. These unit-cell parameters are in agreement with the pressure–volume trend observed, but peak broadening and loss of reflections at high angles may reflect the onset of phase instability on decompression.

## Synthesis and crystallization   

The synthesis of Co_12_P_7_ was performed at high pressures and temperatures in a laser-heated diamond anvil cell (LHDAC). Two samples were loaded for this study in which Co_12_P_7_ was synthesized at 26.9 (8) GPa and 1740 (110) K and 48.2 (5) GPa and 1790 (200) K, respectively. Pressure was generated in BX-90-type (70° angular opening) diamond anvil cells (DACs) with 300 µm culet, Boehler–Almax type diamonds and seats. Co–P samples and a ruby sphere for pressure calibration were loaded into a sample chamber drilled from a rhenium gasket. The chamber was subsequently filled with compressed neon gas (Rivers *et al.*, 2008 ▸). Pressure was determined using the ruby fluorescence scale and the Ne equation of state (Mao & Bell, 1976 ▸; Fei *et al.*, 2007 ▸).

Samples were heated from both sides with 100W Yb-doped fiber lasers at beamline 13-ID-D (GeoSoilEnviroCARS) of the Advanced Photon Source (APS), Argonne National Laboratory. Heating cycles typically lasted ∼15 minutes at target temperatures prior to quench. The lasers were shaped with ∼15 µm flat tops and temperature was measured spectroradiometrically from a 6 µm central region of the laser heated spot using a gray body approximation (Heinz & Jeanloz, 1987 ▸). Axial temperature gradients through the sample were accounted for by applying a 3% correction on temperature measurements (Campbell *et al.*, 2007 ▸, 2009 ▸).

Upon quench from high temperatures, high-pressure samples consisted of agglomerates of Co_12_P_7_ and *Pnma* Co_2_P (Rundqvist, 1960 ▸) crystals of variable grain sizes up to ∼5 µm in diameter. Grains of target phases were identified in reciprocal space and sorted out from the scattering contribution of other grains, neon and diamond. Diffraction data were processed using Dioptas (Prescher & Prakapenka, 2015 ▸) and *CrysAlis Pro* (Rigaku OD, 2018 ▸). Decompression data were collected for both samples in two experimental stations; here we report two selected refinements of the Co_12_P_7_ structure at 48.2 (5) GPa and 15.4 (2) GPa.

## Refinement   

Crystal data, data collection and structure refinement details at 48 GPa and 15 GPa are summarized in Table 2 ▸.

Monochromatic X-ray diffraction measurements took place at beamlines 13-ID-D (2 µm x 3 µm beam, λ = 0.2952 Å) and 13-BM-D (5 µm × 8 µm beam, λ = 0.3344 Å) at APS (Table 2 ▸). Diffraction measurements were collected at synthesis pressures and upon decompression. At target pressure steps, 10 x 10 µm still image maps were collected in 2 µm steps around the heated region. At selected map locations exhibiting the largest crystallites, rotation images were collected spanning ±30° at a rate of 1s per 0.5° step.

Grains of Co_12_P_7_ identified in reciprocal space were indexed to a primitive hexa­gonal lattice. Analysis of systematic absences indicated space group *P* 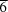 with *Z* = 1. Two grains from distinct loadings and measured at different beamlines were selected for structural refinements as they showed the largest number of observed reflections and good statistical parameters (Table 2 ▸). Structure factors measured in microdiffraction in the LHDAC show some well-known limitations, such as limited resolution and redundancy, reflections overlapped by parasitic scattering, diamond diffraction (Loveday *et al.*, 1990 ▸) and, more notably, variable volume of illuminated crystal during rotation. As could be expected, we identified eight and five outlier reflections in the refinements for the 48 GPa and 15 GPa data sets, respectively, and omitted them in the final calculations. Based on the ratio ‘observed reflections/refined parameters’ and statistical tests (Hamilton, 1965 ▸), we concluded that the P sites should be refined with isotropic displacement parameters (*U*_iso_) whereas the Co sites could be refined with anisotropic displacement parameters. After convergence, site occupancies of Co atoms and P atoms were released in alternate runs. Within uncertainty (< 1.2% for Co and < 1.3% for P), all sites are fully occupied.

## Supplementary Material

Crystal structure: contains datablock(s) Co12P7_at_48GPa, Co12P7_at_15GPa. DOI: 10.1107/S2056989020012657/wm5583sup1.cif

Structure factors: contains datablock(s) Co12P7_at_48GPa. DOI: 10.1107/S2056989020012657/wm5583Co12P7_at_48GPasup2.hkl

Structure factors: contains datablock(s) Co12P7_at_15GPa. DOI: 10.1107/S2056989020012657/wm5583Co12P7_at_15GPasup3.hkl

CCDC references: 2032430, 2032429

Additional supporting information:  crystallographic information; 3D view; checkCIF report

## Figures and Tables

**Table 1:** Selected structural parameters for Co_12_P_7_ at 48 GPa

Group	Maximal bond length (Å)	minimal bond length (Å)	Polyhedron volume (Å^3^)	Distortion index
CoP_4_ (Co0—P4, —P5, —P6)	2.102 (2)	2.063 (2)	4.5433	0.00656
CoP_5_ (Co1—P4, —P5)	2.220 (4)	2.147 (4)	8.1257	0.01085
CoP_5_ (Co2—P4, —P5, —P6)	2.317 (2)	2.197 (4)	9.0766	0.01432
CoP_5_ (Co3—P4, —P5)	2.219 (3)	2.194 (3)	8.3239	0.00514

**Table 2:** Experimental details

	48 GPa	15 GPa
Crystal data		
Chemical formula	Co_12_P_7_	Co_12_P_7_
*M*_r_	923.95	923.95
Crystal system, space group	Hexagonal, *P* 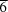	Hexagonal, *P* 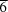
Temperature (K)	293	293
*a*, *c* (Å)	7.9700 (14), 3.2034 (4)	8.253 (5), 3.2902 (18)
*V* (Å^3^)	176.22 (7)	194.1 (3)
*Z*	1	1
Radiation type	Synchrotron, λ = 0.29521 Å	Synchrotron, λ = 0.3344 Å
μ (mm^−1^)	2.47	3.17
Crystal size (mm)	0.01 × 0.01 × 0.01	0.01 × 0.01 × 0.01

Data collection		
Diffractometer	13IDD @ APS	13BMD @ APS
Absorption correction	Multi-scan (*CrysAlis PRO*; Rigaku OD, 2018 ▸)	Multi-scan (*CrysAlis PRO*; Rigaku OD, 2018 ▸)
*T*_min_, *T*_max_	0.789, 1.000	0.546, 1.000
No. of measured, independent and observed [*I* > 2σ(*I*)] reflections	336, 292, 279	592, 321, 253
*R*_int_	0.006	0.055
(sin θ/λ)_max_ (Å^−1^)	0.874	0.762

Refinement		
*R*[*F* ^2^ > 2σ(*F* ^2^)], *wR*(*F* ^2^), *S*	0.037, 0.096, 1.12	0.053, 0.105, 1.11
No. of reflections	292	321
No. of parameters	32	32
Δρ_max_, Δρ_min_ (e Å^−3^)	2.35, −1.81	1.70, −1.74
Absolute structure	Flack *x* determined using 75 quotients [(*I* ^+^)−(*I* ^−^)]/[(*I* ^+^)+(*I* ^−^)] (Parsons *et al.*, 2013 ▸)	Flack *x* determined using 78 quotients [(*I* ^+^)−(*I* ^−^)]/[(*I* ^+^)+(*I* ^−^)] (Parsons *et al.*, 2013 ▸)
Absolute structure parameter	0.42 (6)	0.4 (2)

Computer programs: *CrysAlis PRO* (Rigaku OD, 2018 ▸), *SHELXT* (Sheldrick, 2015*a* ▸), *SHELXL2014/7* (Sheldrick, 2015*b* ▸), *VESTA* (Momma & Izumi, 2011 ▸) and *publCIF* (Westrip, 2010 ▸).

## supplementary crystallographic information

### Dodecacobalt heptaphosphide (Co12P7_at_48GPa) Crystal data

**Table:**

Co_12_P_7_	*D*_x_ = 8.706 Mg m^−^^3^
*M**_r_* = 923.95	Synchrotron radiation, λ = 0.29521 Å
Hexagonal, *P*6	Cell parameters from 292 reflections
*a* = 7.9700 (14) Å	θ = 2.3–14.9°
*c* = 3.2034 (4) Å	µ = 2.47 mm^−^^1^
*V* = 176.22 (7) Å^3^	*T* = 293 K
*Z* = 1	Irregular, black
*F*(000) = 429	0.01 × 0.01 × 0.01 mm

### Dodecacobalt heptaphosphide (Co12P7_at_48GPa) Data collection

**Table:**

13IDD @ APS diffractometer	279 reflections with *I* > 2σ(*I*)
Radiation source: synchrotron	*R*_int_ = 0.006
ω scans	θ_max_ = 15.0°, θ_min_ = 2.1°
Absorption correction: multi-scan (*CrysAlisPro*; Rigaku OD, 2018)	*h* = −6→8
*T*_min_ = 0.789, *T*_max_ = 1.000	*k* = −10→9
336 measured reflections	*l* = −5→5
292 independent reflections	

### Dodecacobalt heptaphosphide (Co12P7_at_48GPa) Refinement

**Table:**

Refinement on *F*^2^	0 restraints
Least-squares matrix: full	*w* = 1/[σ^2^(*F*_o_^2^) + (0.0802*P*)^2^] where *P* = (*F*_o_^2^ + 2*F*_c_^2^)/3
*R*[*F*^2^ > 2σ(*F*^2^)] = 0.037	(Δ/σ)_max_ < 0.001
*wR*(*F*^2^) = 0.096	Δρ_max_ = 2.35 e Å^−^^3^
*S* = 1.12	Δρ_min_ = −1.81 e Å^−^^3^
292 reflections	Absolute structure: Flack *x* determined using 75 quotients [(*I*^+^)-(*I*^-^)]/[(*I*^+^)+(*I*^-^)] (Parsons *et al.*, 2013)
32 parameters	Absolute structure parameter: 0.42 (6)

### Dodecacobalt heptaphosphide (Co12P7_at_48GPa) Special details

**Table:**

Geometry. All esds (except the esd in the dihedral angle between two l.s. planes) are estimated using the full covariance matrix. The cell esds are taken into account individually in the estimation of esds in distances, angles and torsion angles; correlations between esds in cell parameters are only used when they are defined by crystal symmetry. An approximate (isotropic) treatment of cell esds is used for estimating esds involving l.s. planes.

### Dodecacobalt heptaphosphide (Co12P7_at_48GPa) Fractional atomic coordinates and isotropic or equivalent isotropic displacement parameters (Å^2^)

**Table:**

	*x*	*y*	*z*	*U*_iso_*/*U*_eq_	
Co0	0.0185 (3)	0.2676 (3)	0.0000	0.0047 (4)	
Co1	0.1313 (3)	0.6239 (3)	0.0000	0.0047 (4)	
Co2	0.2161 (3)	0.2037 (4)	0.5000	0.0071 (4)	
Co3	0.5185 (3)	0.1341 (3)	0.5000	0.0051 (4)	
P4	0.1693 (5)	0.4529 (5)	0.5000	0.0062 (6)*	
P5	0.4454 (5)	0.2795 (6)	0.0000	0.0050 (6)*	
P6	0.0000	0.0000	0.0000	0.0069 (9)*	

### Dodecacobalt heptaphosphide (Co12P7_at_48GPa) Atomic displacement parameters (Å^2^)

**Table:**

	*U*^11^	*U*^22^	*U*^33^	*U*^12^	*U*^13^	*U*^23^
Co0	0.0044 (8)	0.0030 (8)	0.0062 (5)	0.0015 (7)	0.000	0.000
Co1	0.0038 (8)	0.0024 (7)	0.0071 (8)	0.0009 (6)	0.000	0.000
Co2	0.0081 (8)	0.0069 (9)	0.0079 (6)	0.0049 (7)	0.000	0.000
Co3	0.0038 (8)	0.0028 (8)	0.0069 (7)	0.0005 (6)	0.000	0.000

### Dodecacobalt heptaphosphide (Co12P7_at_48GPa) Geometric parameters (Å, º)

**Table:**

Co0—P6	2.0629 (18)	Co2—Co3	2.730 (2)
Co0—P5^i^	2.091 (4)	Co3—P5^xi^	2.194 (3)
Co0—P4	2.102 (2)	Co3—P5^xii^	2.194 (3)
Co0—P4^ii^	2.102 (2)	Co3—P4^viii^	2.197 (5)
Co0—Co3^iii^	2.458 (2)	Co3—P5^vii^	2.218 (3)
Co0—Co3^i^	2.458 (2)	Co3—P5	2.219 (3)
Co0—Co2^ii^	2.4710 (19)	Co3—Co0^viii^	2.458 (2)
Co0—Co2	2.4710 (19)	Co3—Co0^ix^	2.458 (2)
Co0—Co2^iii^	2.497 (2)	Co3—Co3^xii^	2.474 (3)
Co0—Co2^i^	2.497 (2)	Co3—Co3^xiii^	2.475 (3)
Co0—Co1	2.514 (3)	Co3—Co1^viii^	2.578 (2)
Co0—Co1^iv^	2.515 (2)	Co3—Co1^ix^	2.578 (2)
Co1—P5^v^	2.147 (4)	P4—Co0^vii^	2.102 (2)
Co1—P4^v^	2.180 (3)	P4—Co1^x^	2.180 (3)
Co1—P4^vi^	2.180 (3)	P4—Co1^iv^	2.180 (3)
Co1—P4^ii^	2.220 (3)	P4—Co3^i^	2.197 (5)
Co1—P4	2.220 (3)	P4—Co1^vii^	2.220 (3)
Co1—Co0^v^	2.515 (2)	P4—P4^iv^	2.674 (6)
Co1—Co1^v^	2.546 (3)	P4—P4^v^	2.674 (6)
Co1—Co1^iv^	2.546 (3)	P5—Co0^viii^	2.091 (4)
Co1—Co3^iii^	2.578 (2)	P5—Co1^iv^	2.147 (4)
Co1—Co3^i^	2.578 (2)	P5—Co3^xiii^	2.194 (3)
Co1—Co2^v^	2.639 (2)	P5—Co3^xiv^	2.194 (3)
Co2—P4	2.197 (4)	P5—Co3^ii^	2.218 (3)
Co2—P5	2.273 (3)	P5—Co2^ii^	2.273 (3)
Co2—P5^vii^	2.273 (3)	P6—Co0^i^	2.0629 (18)
Co2—P6	2.3174 (17)	P6—Co0^viii^	2.0629 (18)
Co2—P6^vii^	2.3174 (17)	P6—Co2^xv^	2.3174 (17)
Co2—Co0^vii^	2.4710 (19)	P6—Co2^viii^	2.3174 (17)
Co2—Co0^viii^	2.497 (2)	P6—Co2^ii^	2.3174 (17)
Co2—Co0^ix^	2.497 (2)	P6—Co2^iii^	2.3175 (17)
Co2—Co1^x^	2.639 (2)	P6—Co2^i^	2.3175 (17)
Co2—Co1^iv^	2.639 (2)		
			
P6—Co0—P5^i^	96.84 (13)	P4—Co2—Co3	138.57 (14)
P6—Co0—P4	116.54 (11)	P5—Co2—Co3	51.66 (8)
P5^i^—Co0—P4	114.33 (11)	P5^vii^—Co2—Co3	51.66 (8)
P6—Co0—P4^ii^	116.54 (11)	P6—Co2—Co3	106.26 (8)
P5^i^—Co0—P4^ii^	114.33 (11)	P6^vii^—Co2—Co3	106.26 (8)
P4—Co0—P4^ii^	99.31 (14)	Co0^vii^—Co2—Co3	139.47 (4)
P6—Co0—Co3^iii^	126.74 (6)	Co0—Co2—Co3	139.47 (4)
P5^i^—Co0—Co3^iii^	57.69 (9)	Co0^viii^—Co2—Co3	55.89 (7)
P4—Co0—Co3^iii^	116.61 (12)	Co0^ix^—Co2—Co3	55.89 (7)
P4^ii^—Co0—Co3^iii^	56.96 (11)	Co1^x^—Co2—Co3	96.63 (8)
P6—Co0—Co3^i^	126.74 (6)	Co1^iv^—Co2—Co3	96.63 (8)
P5^i^—Co0—Co3^i^	57.69 (9)	P5^xi^—Co3—P5^xii^	93.78 (15)
P4—Co0—Co3^i^	56.96 (11)	P5^xi^—Co3—P4^viii^	107.43 (14)
P4^ii^—Co0—Co3^i^	116.61 (12)	P5^xii^—Co3—P4^viii^	107.43 (14)
Co3^iii^—Co0—Co3^i^	81.31 (9)	P5^xi^—Co3—P5^vii^	77.38 (14)
P6—Co0—Co2^ii^	60.69 (6)	P5^xii^—Co3—P5^vii^	146.69 (16)
P5^i^—Co0—Co2^ii^	129.60 (8)	P4^viii^—Co3—P5^vii^	105.86 (14)
P4—Co0—Co2^ii^	116.07 (13)	P5^xi^—Co3—P5	146.69 (16)
P4^ii^—Co0—Co2^ii^	56.73 (11)	P5^xii^—Co3—P5	77.38 (14)
Co3^iii^—Co0—Co2^ii^	98.20 (5)	P4^viii^—Co3—P5	105.86 (14)
Co3^i^—Co0—Co2^ii^	170.85 (9)	P5^vii^—Co3—P5	92.44 (15)
P6—Co0—Co2	60.69 (6)	P5^xi^—Co3—Co0^viii^	160.35 (15)
P5^i^—Co0—Co2	129.60 (8)	P5^xii^—Co3—Co0^viii^	89.46 (9)
P4—Co0—Co2	56.73 (11)	P4^viii^—Co3—Co0^viii^	53.31 (7)
P4^ii^—Co0—Co2	116.07 (13)	P5^vii^—Co3—Co0^viii^	109.66 (12)
Co3^iii^—Co0—Co2	170.85 (9)	P5—Co3—Co0^viii^	52.82 (11)
Co3^i^—Co0—Co2	98.20 (5)	P5^xi^—Co3—Co0^ix^	89.46 (9)
Co2^ii^—Co0—Co2	80.81 (8)	P5^xii^—Co3—Co0^ix^	160.35 (15)
P6—Co0—Co2^iii^	60.19 (6)	P4^viii^—Co3—Co0^ix^	53.31 (7)
P5^i^—Co0—Co2^iii^	58.60 (11)	P5^vii^—Co3—Co0^ix^	52.82 (11)
P4—Co0—Co2^iii^	169.97 (10)	P5—Co3—Co0^ix^	109.66 (12)
P4^ii^—Co0—Co2^iii^	90.40 (8)	Co0^viii^—Co3—Co0^ix^	81.32 (9)
Co3^iii^—Co0—Co2^iii^	66.85 (7)	P5^xi^—Co3—Co3^xii^	56.36 (10)
Co3^i^—Co0—Co2^iii^	116.27 (10)	P5^xii^—Co3—Co3^xii^	56.36 (10)
Co2^ii^—Co0—Co2^iii^	71.44 (10)	P4^viii^—Co3—Co3^xii^	151.84 (16)
Co2—Co0—Co2^iii^	120.88 (10)	P5^vii^—Co3—Co3^xii^	93.38 (11)
P6—Co0—Co2^i^	60.19 (6)	P5—Co3—Co3^xii^	93.38 (11)
P5^i^—Co0—Co2^i^	58.60 (11)	Co0^viii^—Co3—Co3^xii^	138.36 (5)
P4—Co0—Co2^i^	90.40 (8)	Co0^ix^—Co3—Co3^xii^	138.36 (5)
P4^ii^—Co0—Co2^i^	169.97 (10)	P5^xi^—Co3—Co3^xiii^	93.99 (11)
Co3^iii^—Co0—Co2^i^	116.27 (10)	P5^xii^—Co3—Co3^xiii^	93.99 (11)
Co3^i^—Co0—Co2^i^	66.85 (7)	P4^viii^—Co3—Co3^xiii^	148.16 (16)
Co2^ii^—Co0—Co2^i^	120.88 (10)	P5^vii^—Co3—Co3^xiii^	55.42 (11)
Co2—Co0—Co2^i^	71.44 (10)	P5—Co3—Co3^xiii^	55.42 (11)
Co2^iii^—Co0—Co2^i^	79.79 (9)	Co0^viii^—Co3—Co3^xiii^	105.12 (10)
P6—Co0—Co1	165.52 (9)	Co0^ix^—Co3—Co3^xiii^	105.12 (10)
P5^i^—Co0—Co1	97.64 (13)	Co3^xii^—Co3—Co3^xiii^	60.0
P4—Co0—Co1	56.65 (9)	P5^xi^—Co3—Co1^viii^	107.55 (12)
P4^ii^—Co0—Co1	56.65 (9)	P5^xii^—Co3—Co1^viii^	52.72 (10)
Co3^iii^—Co0—Co1	62.44 (6)	P4^viii^—Co3—Co1^viii^	54.71 (8)
Co3^i^—Co0—Co1	62.44 (6)	P5^vii^—Co3—Co1^viii^	160.55 (15)
Co2^ii^—Co0—Co1	109.16 (7)	P5—Co3—Co1^viii^	92.62 (8)
Co2—Co0—Co1	109.16 (7)	Co0^viii^—Co3—Co1^viii^	59.83 (7)
Co2^iii^—Co0—Co1	128.86 (7)	Co0^ix^—Co3—Co1^viii^	107.89 (10)
Co2^i^—Co0—Co1	128.86 (7)	Co3^xii^—Co3—Co1^viii^	105.04 (11)
P6—Co0—Co1^iv^	104.70 (9)	Co3^xiii^—Co3—Co1^viii^	140.36 (4)
P5^i^—Co0—Co1^iv^	158.46 (14)	P5^xi^—Co3—Co1^ix^	52.72 (10)
P4—Co0—Co1^iv^	55.49 (9)	P5^xii^—Co3—Co1^ix^	107.55 (12)
P4^ii^—Co0—Co1^iv^	55.49 (9)	P4^viii^—Co3—Co1^ix^	54.71 (8)
Co3^iii^—Co0—Co1^iv^	107.42 (7)	P5^vii^—Co3—Co1^ix^	92.62 (8)
Co3^i^—Co0—Co1^iv^	107.42 (7)	P5—Co3—Co1^ix^	160.55 (15)
Co2^ii^—Co0—Co1^iv^	63.90 (7)	Co0^viii^—Co3—Co1^ix^	107.89 (10)
Co2—Co0—Co1^iv^	63.90 (7)	Co0^ix^—Co3—Co1^ix^	59.83 (7)
Co2^iii^—Co0—Co1^iv^	133.74 (7)	Co3^xii^—Co3—Co1^ix^	105.04 (11)
Co2^i^—Co0—Co1^iv^	133.74 (7)	Co3^xiii^—Co3—Co1^ix^	140.36 (4)
Co1—Co0—Co1^iv^	60.82 (8)	Co1^viii^—Co3—Co1^ix^	76.82 (8)
P5^v^—Co1—P4^v^	108.68 (12)	P5^xi^—Co3—Co2	130.38 (8)
P5^v^—Co1—P4^vi^	108.68 (12)	P5^xii^—Co3—Co2	130.38 (8)
P4^v^—Co1—P4^vi^	94.55 (18)	P4^viii^—Co3—Co2	82.57 (13)
P5^v^—Co1—P4^ii^	108.29 (12)	P5^vii^—Co3—Co2	53.49 (10)
P4^v^—Co1—P4^ii^	143.01 (13)	P5—Co3—Co2	53.49 (10)
P4^vi^—Co1—P4^ii^	74.85 (14)	Co0^viii^—Co3—Co2	57.26 (7)
P5^v^—Co1—P4	108.29 (12)	Co0^ix^—Co3—Co2	57.26 (7)
P4^v^—Co1—P4	74.85 (14)	Co3^xii^—Co3—Co2	125.59 (11)
P4^vi^—Co1—P4	143.01 (13)	Co3^xiii^—Co3—Co2	65.59 (11)
P4^ii^—Co1—P4	92.36 (16)	Co1^viii^—Co3—Co2	116.75 (8)
P5^v^—Co1—Co0	89.08 (13)	Co1^ix^—Co3—Co2	116.75 (8)
P4^v^—Co1—Co0	127.10 (9)	Co0—P4—Co0^vii^	99.31 (14)
P4^vi^—Co1—Co0	127.10 (9)	Co0—P4—Co1^x^	144.1 (2)
P4^ii^—Co1—Co0	52.26 (8)	Co0^vii^—P4—Co1^x^	71.92 (7)
P4—Co1—Co0	52.26 (8)	Co0—P4—Co1^iv^	71.92 (7)
P5^v^—Co1—Co0^v^	91.74 (12)	Co0^vii^—P4—Co1^iv^	144.1 (2)
P4^v^—Co1—Co0^v^	52.58 (9)	Co1^x^—P4—Co1^iv^	94.55 (17)
P4^vi^—Co1—Co0^v^	52.58 (9)	Co0—P4—Co2	70.15 (11)
P4^ii^—Co1—Co0^v^	127.40 (9)	Co0^vii^—P4—Co2	70.15 (11)
P4—Co1—Co0^v^	127.40 (9)	Co1^x^—P4—Co2	74.16 (12)
Co0—Co1—Co0^v^	179.18 (8)	Co1^iv^—P4—Co2	74.16 (12)
P5^v^—Co1—Co1^v^	151.31 (16)	Co0—P4—Co3^i^	69.73 (11)
P4^v^—Co1—Co1^v^	55.38 (11)	Co0^vii^—P4—Co3^i^	69.73 (11)
P4^vi^—Co1—Co1^v^	55.38 (11)	Co1^x^—P4—Co3^i^	132.68 (9)
P4^ii^—Co1—Co1^v^	91.21 (10)	Co1^iv^—P4—Co3^i^	132.68 (9)
P4—Co1—Co1^v^	91.21 (10)	Co2—P4—Co3^i^	116.01 (16)
Co0—Co1—Co1^v^	119.61 (8)	Co0—P4—Co1	71.09 (7)
Co0^v^—Co1—Co1^v^	59.56 (10)	Co0^vii^—P4—Co1	140.9 (2)
P5^v^—Co1—Co1^iv^	148.69 (16)	Co1^x^—P4—Co1	136.84 (16)
P4^v^—Co1—Co1^iv^	92.13 (10)	Co1^iv^—P4—Co1	70.69 (9)
P4^vi^—Co1—Co1^iv^	92.13 (10)	Co2—P4—Co1	133.80 (8)
P4^ii^—Co1—Co1^iv^	53.93 (10)	Co3^i^—P4—Co1	71.42 (12)
P4—Co1—Co1^iv^	53.93 (10)	Co0—P4—Co1^vii^	140.9 (2)
Co0—Co1—Co1^iv^	59.61 (8)	Co0^vii^—P4—Co1^vii^	71.09 (7)
Co0^v^—Co1—Co1^iv^	119.56 (10)	Co1^x^—P4—Co1^vii^	70.69 (9)
Co1^v^—Co1—Co1^iv^	60.0	Co1^iv^—P4—Co1^vii^	136.84 (17)
P5^v^—Co1—Co3^iii^	54.42 (8)	Co2—P4—Co1^vii^	133.80 (8)
P4^v^—Co1—Co3^iii^	163.07 (13)	Co3^i^—P4—Co1^vii^	71.42 (12)
P4^vi^—Co1—Co3^iii^	92.51 (9)	Co1—P4—Co1^vii^	92.36 (16)
P4^ii^—Co1—Co3^iii^	53.87 (11)	Co0—P4—P4^iv^	125.14 (14)
P4—Co1—Co3^iii^	107.88 (12)	Co0^vii^—P4—P4^iv^	125.14 (14)
Co0—Co1—Co3^iii^	57.72 (8)	Co1^x^—P4—P4^iv^	53.25 (12)
Co0^v^—Co1—Co3^iii^	122.84 (8)	Co1^iv^—P4—P4^iv^	53.25 (12)
Co1^v^—Co1—Co3^iii^	139.67 (4)	Co2—P4—P4^iv^	94.4 (2)
Co1^iv^—Co1—Co3^iii^	102.97 (11)	Co3^i^—P4—P4^iv^	149.6 (2)
P5^v^—Co1—Co3^i^	54.42 (8)	Co1—P4—P4^iv^	87.91 (10)
P4^v^—Co1—Co3^i^	92.51 (9)	Co1^vii^—P4—P4^iv^	87.91 (10)
P4^vi^—Co1—Co3^i^	163.07 (13)	Co0—P4—P4^v^	122.98 (14)
P4^ii^—Co1—Co3^i^	107.88 (12)	Co0^vii^—P4—P4^v^	122.98 (14)
P4—Co1—Co3^i^	53.87 (11)	Co1^x^—P4—P4^v^	88.73 (10)
Co0—Co1—Co3^i^	57.72 (8)	Co1^iv^—P4—P4^v^	88.73 (10)
Co0^v^—Co1—Co3^i^	122.84 (8)	Co2—P4—P4^v^	154.4 (2)
Co1^v^—Co1—Co3^i^	139.67 (4)	Co3^i^—P4—P4^v^	89.6 (2)
Co1^iv^—Co1—Co3^i^	102.97 (11)	Co1—P4—P4^v^	51.90 (11)
Co3^iii^—Co1—Co3^i^	76.82 (8)	Co1^vii^—P4—P4^v^	51.90 (11)
P5^v^—Co1—Co2^v^	55.58 (8)	P4^iv^—P4—P4^v^	60.0
P4^v^—Co1—Co2^v^	53.20 (10)	Co0^viii^—P5—Co1^iv^	126.72 (19)
P4^vi^—Co1—Co2^v^	107.02 (11)	Co0^viii^—P5—Co3^xiii^	132.10 (9)
P4^ii^—Co1—Co2^v^	163.76 (12)	Co1^iv^—P5—Co3^xiii^	72.86 (12)
P4—Co1—Co2^v^	94.83 (9)	Co0^viii^—P5—Co3^xiv^	132.10 (9)
Co0—Co1—Co2^v^	123.33 (7)	Co1^iv^—P5—Co3^xiv^	72.86 (12)
Co0^v^—Co1—Co2^v^	57.23 (6)	Co3^xiii^—P5—Co3^xiv^	93.78 (15)
Co1^v^—Co1—Co2^v^	103.16 (10)	Co0^viii^—P5—Co3^ii^	69.48 (13)
Co1^iv^—Co1—Co2^v^	140.65 (5)	Co1^iv^—P5—Co3^ii^	133.45 (8)
Co3^iii^—Co1—Co2^v^	109.96 (9)	Co3^xiii^—P5—Co3^ii^	133.08 (18)
Co3^i^—Co1—Co2^v^	65.62 (7)	Co3^xiv^—P5—Co3^ii^	68.22 (10)
P4—Co2—P5	103.71 (12)	Co0^viii^—P5—Co3	69.48 (13)
P4—Co2—P5^vii^	103.71 (12)	Co1^iv^—P5—Co3	133.45 (8)
P5—Co2—P5^vii^	89.59 (15)	Co3^xiii^—P5—Co3	68.22 (10)
P4—Co2—P6	103.35 (9)	Co3^xiv^—P5—Co3	133.08 (18)
P5—Co2—P6	85.20 (8)	Co3^ii^—P5—Co3	92.44 (15)
P5^vii^—Co2—P6	152.92 (13)	Co0^viii^—P5—Co2	69.66 (13)
P4—Co2—P6^vii^	103.35 (9)	Co1^iv^—P5—Co2	73.26 (11)
P5—Co2—P6^vii^	152.92 (13)	Co3^xiii^—P5—Co2	78.50 (8)
P5^vii^—Co2—P6^vii^	85.20 (8)	Co3^xiv^—P5—Co2	146.03 (19)
P6—Co2—P6^vii^	87.44 (8)	Co3^ii^—P5—Co2	139.1 (2)
P4—Co2—Co0^vii^	53.12 (8)	Co3—P5—Co2	74.85 (8)
P5—Co2—Co0^vii^	155.85 (14)	Co0^viii^—P5—Co2^ii^	69.66 (13)
P5^vii^—Co2—Co0^vii^	89.95 (7)	Co1^iv^—P5—Co2^ii^	73.25 (11)
P6—Co2—Co0^vii^	105.40 (8)	Co3^xiii^—P5—Co2^ii^	146.03 (19)
P6^vii^—Co2—Co0^vii^	50.91 (5)	Co3^xiv^—P5—Co2^ii^	78.50 (8)
P4—Co2—Co0	53.12 (8)	Co3^ii^—P5—Co2^ii^	74.85 (8)
P5—Co2—Co0	89.95 (7)	Co3—P5—Co2^ii^	139.1 (2)
P5^vii^—Co2—Co0	155.85 (14)	Co2—P5—Co2^ii^	89.59 (15)
P6—Co2—Co0	50.91 (5)	Co0^i^—P6—Co0	120.0
P6^vii^—Co2—Co0	105.40 (8)	Co0^i^—P6—Co0^viii^	120.0
Co0^vii^—Co2—Co0	80.81 (8)	Co0—P6—Co0^viii^	120.0
P4—Co2—Co0^viii^	140.08 (4)	Co0^i^—P6—Co2^xv^	69.24 (6)
P5—Co2—Co0^viii^	51.74 (11)	Co0—P6—Co2^xv^	136.27 (4)
P5^vii^—Co2—Co0^viii^	106.53 (11)	Co0^viii^—P6—Co2^xv^	68.40 (6)
P6—Co2—Co0^viii^	50.57 (5)	Co0^i^—P6—Co2^viii^	69.24 (6)
P6^vii^—Co2—Co0^viii^	104.56 (10)	Co0—P6—Co2^viii^	136.27 (4)
Co0^vii^—Co2—Co0^viii^	149.98 (11)	Co0^viii^—P6—Co2^viii^	68.40 (6)
Co0—Co2—Co0^viii^	91.97 (7)	Co2^xv^—P6—Co2^viii^	87.45 (8)
P4—Co2—Co0^ix^	140.08 (4)	Co0^i^—P6—Co2	136.27 (4)
P5—Co2—Co0^ix^	106.54 (11)	Co0—P6—Co2	68.40 (6)
P5^vii^—Co2—Co0^ix^	51.74 (11)	Co0^viii^—P6—Co2	69.24 (6)
P6—Co2—Co0^ix^	104.56 (10)	Co2^xv^—P6—Co2	137.63 (3)
P6^vii^—Co2—Co0^ix^	50.57 (5)	Co2^viii^—P6—Co2	77.49 (6)
Co0^vii^—Co2—Co0^ix^	91.97 (7)	Co0^i^—P6—Co2^ii^	136.27 (4)
Co0—Co2—Co0^ix^	149.98 (11)	Co0—P6—Co2^ii^	68.40 (6)
Co0^viii^—Co2—Co0^ix^	79.79 (9)	Co0^viii^—P6—Co2^ii^	69.24 (6)
P4—Co2—Co1^x^	52.64 (9)	Co2^xv^—P6—Co2^ii^	77.49 (6)
P5—Co2—Co1^x^	103.19 (12)	Co2^viii^—P6—Co2^ii^	137.64 (3)
P5^vii^—Co2—Co1^x^	51.16 (10)	Co2—P6—Co2^ii^	87.44 (8)
P6—Co2—Co1^x^	155.65 (9)	Co0^i^—P6—Co2^iii^	68.40 (6)
P6^vii^—Co2—Co1^x^	94.13 (4)	Co0—P6—Co2^iii^	69.24 (6)
Co0^vii^—Co2—Co1^x^	58.87 (6)	Co0^viii^—P6—Co2^iii^	136.27 (4)
Co0—Co2—Co1^x^	105.65 (9)	Co2^xv^—P6—Co2^iii^	77.49 (6)
Co0^viii^—Co2—Co1^x^	149.97 (10)	Co2^viii^—P6—Co2^iii^	137.63 (3)
Co0^ix^—Co2—Co1^x^	95.00 (6)	Co2—P6—Co2^iii^	137.63 (3)
P4—Co2—Co1^iv^	52.64 (9)	Co2^ii^—P6—Co2^iii^	77.49 (6)
P5—Co2—Co1^iv^	51.16 (10)	Co0^i^—P6—Co2^i^	68.40 (6)
P5^vii^—Co2—Co1^iv^	103.19 (13)	Co0—P6—Co2^i^	69.24 (6)
P6—Co2—Co1^iv^	94.13 (4)	Co0^viii^—P6—Co2^i^	136.27 (4)
P6^vii^—Co2—Co1^iv^	155.65 (9)	Co2^xv^—P6—Co2^i^	137.63 (3)
Co0^vii^—Co2—Co1^iv^	105.65 (9)	Co2^viii^—P6—Co2^i^	77.49 (6)
Co0—Co2—Co1^iv^	58.87 (6)	Co2—P6—Co2^i^	77.49 (6)
Co0^viii^—Co2—Co1^iv^	95.00 (6)	Co2^ii^—P6—Co2^i^	137.63 (3)
Co0^ix^—Co2—Co1^iv^	149.97 (10)	Co2^iii^—P6—Co2^i^	87.44 (8)
Co1^x^—Co2—Co1^iv^	74.74 (7)		

Symmetry codes: (i) −*y*, *x*−*y*, *z*; (ii) *x*, *y*, *z*−1; (iii) −*y*, *x*−*y*, *z*−1; (iv) −*y*+1, *x*−*y*+1, *z*; (v) −*x*+*y*, −*x*+1, *z*; (vi) −*x*+*y*, −*x*+1, *z*−1; (vii) *x*, *y*, *z*+1; (viii) −*x*+*y*, −*x*, *z*; (ix) −*x*+*y*, −*x*, *z*+1; (x) −*y*+1, *x*−*y*+1, *z*+1; (xi) −*y*+1, *x*−*y*, *z*+1; (xii) −*y*+1, *x*−*y*, *z*; (xiii) −*x*+*y*+1, −*x*+1, *z*; (xiv) −*x*+*y*+1, −*x*+1, *z*−1; (xv) −*x*+*y*, −*x*, *z*−1.

### (Co12P7_at_15GPa) Crystal data

**Table:**

Co_12_P_7_	*D*_x_ = 7.905 Mg m^−^^3^
*M**_r_* = 923.95	Synchrotron radiation, λ = 0.3344 Å
Hexagonal, *P*6	Cell parameters from 249 reflections
*a* = 8.253 (5) Å	θ = 2.9–14.7°
*c* = 3.2902 (18) Å	µ = 3.17 mm^−^^1^
*V* = 194.1 (3) Å^3^	*T* = 293 K
*Z* = 1	Irregular, black
*F*(000) = 429	0.01 × 0.01 × 0.01 mm

### (Co12P7_at_15GPa) Data collection

**Table:**

13BMD @ APS diffractometer	253 reflections with *I* > 2σ(*I*)
Radiation source: synchrotron	*R*_int_ = 0.055
/w scan	θ_max_ = 14.8°, θ_min_ = 3.2°
Absorption correction: multi-scan (*CrysAlisPro*; Rigaku OD, 2018)	*h* = −11→12
*T*_min_ = 0.546, *T*_max_ = 1.000	*k* = −9→8
592 measured reflections	*l* = −4→4
321 independent reflections	

### (Co12P7_at_15GPa) Refinement

**Table:**

Refinement on *F*^2^	0 restraints
Least-squares matrix: full	*w* = 1/[σ^2^(*F*_o_^2^) + (0.0219*P*)^2^ + 2.8589*P*] where *P* = (*F*_o_^2^ + 2*F*_c_^2^)/3
*R*[*F*^2^ > 2σ(*F*^2^)] = 0.053	(Δ/σ)_max_ < 0.001
*wR*(*F*^2^) = 0.105	Δρ_max_ = 1.70 e Å^−^^3^
*S* = 1.11	Δρ_min_ = −1.74 e Å^−^^3^
321 reflections	Absolute structure: Flack *x* determined using 78 quotients [(*I*^+^)-(*I*^-^)]/[(*I*^+^)+(*I*^-^)] (Parsons *et al.*, 2013)
32 parameters	Absolute structure parameter: 0.4 (2)

### (Co12P7_at_15GPa) Special details

**Table:**

Geometry. All esds (except the esd in the dihedral angle between two l.s. planes) are estimated using the full covariance matrix. The cell esds are taken into account individually in the estimation of esds in distances, angles and torsion angles; correlations between esds in cell parameters are only used when they are defined by crystal symmetry. An approximate (isotropic) treatment of cell esds is used for estimating esds involving l.s. planes.

### (Co12P7_at_15GPa) Fractional atomic coordinates and isotropic or equivalent isotropic displacement parameters (Å^2^)

**Table:**

	*x*	*y*	*z*	*U*_iso_*/*U*_eq_	
Co0	0.0153 (6)	0.2651 (6)	0.0000	0.0114 (8)	
Co1	0.1320 (7)	0.6234 (7)	0.0000	0.0123 (9)	
Co2	0.2135 (6)	0.2038 (8)	0.5000	0.0151 (9)	
Co3	0.5195 (7)	0.1363 (7)	0.5000	0.0107 (9)	
P4	0.1656 (11)	0.4503 (11)	0.5000	0.0102 (15)*	
P5	0.4425 (12)	0.2809 (13)	0.0000	0.0086 (15)*	
P6	0.0000	0.0000	0.0000	0.012 (3)*	

### (Co12P7_at_15GPa) Atomic displacement parameters (Å^2^)

**Table:**

	*U*^11^	*U*^22^	*U*^33^	*U*^12^	*U*^13^	*U*^23^
Co0	0.014 (2)	0.016 (2)	0.0072 (18)	0.009 (2)	0.000	0.000
Co1	0.019 (2)	0.016 (2)	0.003 (2)	0.0101 (18)	0.000	0.000
Co2	0.024 (2)	0.020 (3)	0.005 (2)	0.014 (2)	0.000	0.000
Co3	0.011 (2)	0.012 (2)	0.008 (2)	0.0052 (16)	0.000	0.000

### (Co12P7_at_15GPa) Geometric parameters (Å, º)

**Table:**

Co0—P6	2.128 (5)	Co2—Co1^iv^	2.731 (5)
Co0—P5^i^	2.148 (10)	Co2—Co3	2.846 (7)
Co0—P4	2.165 (6)	Co3—P5^xi^	2.263 (7)
Co0—P4^ii^	2.165 (6)	Co3—P5^xii^	2.263 (7)
Co0—Co3^iii^	2.538 (5)	Co3—P4^viii^	2.266 (10)
Co0—Co3^i^	2.538 (5)	Co3—P5^vii^	2.301 (8)
Co0—Co2^ii^	2.543 (6)	Co3—P5	2.301 (8)
Co0—Co2	2.543 (6)	Co3—Co3^xiii^	2.536 (9)
Co0—Co2^iii^	2.571 (6)	Co3—Co3^xii^	2.536 (9)
Co0—Co2^i^	2.571 (6)	Co3—Co0^viii^	2.538 (5)
Co0—Co1	2.612 (7)	Co3—Co0^ix^	2.538 (5)
Co0—Co1^iv^	2.634 (7)	Co3—Co1^viii^	2.674 (5)
Co1—P5^v^	2.202 (10)	Co3—Co1^ix^	2.674 (5)
Co1—P4^v^	2.266 (7)	P4—Co0^vii^	2.165 (6)
Co1—P4^vi^	2.266 (7)	P4—Co1^x^	2.266 (7)
Co1—P4^ii^	2.284 (7)	P4—Co1^iv^	2.266 (7)
Co1—P4	2.285 (7)	P4—Co3^i^	2.266 (10)
Co1—Co1^v^	2.624 (9)	P4—Co1^vii^	2.285 (7)
Co1—Co1^iv^	2.624 (9)	P5—Co0^viii^	2.148 (10)
Co1—Co0^v^	2.634 (7)	P5—Co1^iv^	2.202 (10)
Co1—Co3^iii^	2.674 (5)	P5—Co3^xiii^	2.263 (7)
Co1—Co3^i^	2.674 (5)	P5—Co3^xiv^	2.263 (7)
Co1—Co2^v^	2.731 (5)	P5—Co3^ii^	2.301 (8)
Co2—P4	2.258 (9)	P5—Co2^ii^	2.341 (7)
Co2—P5^vii^	2.341 (7)	P6—Co0^i^	2.128 (5)
Co2—P5	2.341 (7)	P6—Co0^viii^	2.128 (5)
Co2—P6	2.383 (4)	P6—Co2^xv^	2.383 (4)
Co2—P6^vii^	2.383 (4)	P6—Co2^iii^	2.383 (4)
Co2—Co0^vii^	2.543 (6)	P6—Co2^i^	2.383 (4)
Co2—Co0^viii^	2.571 (6)	P6—Co2^viii^	2.383 (4)
Co2—Co0^ix^	2.571 (6)	P6—Co2^ii^	2.383 (4)
Co2—Co1^x^	2.731 (5)		
			
P6—Co0—P5^i^	96.9 (3)	P5^vii^—Co2—Co1^iv^	102.3 (3)
P6—Co0—P4	116.4 (2)	P5—Co2—Co1^iv^	50.7 (2)
P5^i^—Co0—P4	114.7 (3)	P6—Co2—Co1^iv^	94.84 (11)
P6—Co0—P4^ii^	116.4 (2)	P6^vii^—Co2—Co1^iv^	156.4 (2)
P5^i^—Co0—P4^ii^	114.7 (3)	Co0^vii^—Co2—Co1^iv^	106.0 (2)
P4—Co0—P4^ii^	98.9 (4)	Co0—Co2—Co1^iv^	59.81 (15)
P6—Co0—Co3^iii^	127.43 (16)	Co0^viii^—Co2—Co1^iv^	94.89 (12)
P5^i^—Co0—Co3^iii^	58.1 (2)	Co0^ix^—Co2—Co1^iv^	148.9 (2)
P4—Co0—Co3^iii^	116.1 (3)	Co1^x^—Co2—Co1^iv^	74.08 (17)
P4^ii^—Co0—Co3^iii^	56.9 (2)	P4—Co2—Co3	138.5 (3)
P6—Co0—Co3^i^	127.43 (16)	P5^vii^—Co2—Co3	51.6 (2)
P5^i^—Co0—Co3^i^	58.1 (2)	P5—Co2—Co3	51.6 (2)
P4—Co0—Co3^i^	56.9 (2)	P6—Co2—Co3	106.1 (2)
P4^ii^—Co0—Co3^i^	116.1 (3)	P6^vii^—Co2—Co3	106.1 (2)
Co3^iii^—Co0—Co3^i^	80.8 (2)	Co0^vii^—Co2—Co3	139.57 (11)
P6—Co0—Co2^ii^	60.57 (16)	Co0—Co2—Co3	139.58 (11)
P5^i^—Co0—Co2^ii^	129.6 (2)	Co0^viii^—Co2—Co3	55.61 (16)
P4—Co0—Co2^ii^	115.7 (3)	Co0^ix^—Co2—Co3	55.61 (16)
P4^ii^—Co0—Co2^ii^	56.6 (2)	Co1^x^—Co2—Co3	95.9 (2)
Co3^iii^—Co0—Co2^ii^	98.44 (12)	Co1^iv^—Co2—Co3	95.9 (2)
Co3^i^—Co0—Co2^ii^	170.2 (3)	P5^xi^—Co3—P5^xii^	93.3 (4)
P6—Co0—Co2	60.57 (16)	P5^xi^—Co3—P4^viii^	106.5 (3)
P5^i^—Co0—Co2	129.6 (2)	P5^xii^—Co3—P4^viii^	106.5 (3)
P4—Co0—Co2	56.6 (2)	P5^xi^—Co3—P5^vii^	79.0 (4)
P4^ii^—Co0—Co2	115.7 (3)	P5^xii^—Co3—P5^vii^	148.1 (4)
Co3^iii^—Co0—Co2	170.2 (3)	P4^viii^—Co3—P5^vii^	105.3 (3)
Co3^i^—Co0—Co2	98.44 (12)	P5^xi^—Co3—P5	148.1 (4)
Co2^ii^—Co0—Co2	80.6 (2)	P5^xii^—Co3—P5	79.0 (4)
P6—Co0—Co2^iii^	60.07 (15)	P4^viii^—Co3—P5	105.3 (3)
P5^i^—Co0—Co2^iii^	58.7 (2)	P5^vii^—Co3—P5	91.3 (4)
P4—Co0—Co2^iii^	170.1 (3)	P5^xi^—Co3—Co3^xiii^	95.1 (3)
P4^ii^—Co0—Co2^iii^	90.7 (2)	P5^xii^—Co3—Co3^xiii^	95.1 (3)
Co3^iii^—Co0—Co2^iii^	67.70 (14)	P4^viii^—Co3—Co3^xiii^	148.1 (4)
Co3^i^—Co0—Co2^iii^	116.8 (2)	P5^vii^—Co3—Co3^xiii^	55.5 (3)
Co2^ii^—Co0—Co2^iii^	71.4 (2)	P5—Co3—Co3^xiii^	55.5 (3)
Co2—Co0—Co2^iii^	120.6 (2)	P5^xi^—Co3—Co3^xii^	57.0 (3)
P6—Co0—Co2^i^	60.07 (15)	P5^xii^—Co3—Co3^xii^	57.0 (3)
P5^i^—Co0—Co2^i^	58.7 (2)	P4^viii^—Co3—Co3^xii^	151.9 (4)
P4—Co0—Co2^i^	90.7 (2)	P5^vii^—Co3—Co3^xii^	94.1 (3)
P4^ii^—Co0—Co2^i^	170.1 (3)	P5—Co3—Co3^xii^	94.1 (3)
Co3^iii^—Co0—Co2^i^	116.8 (2)	Co3^xiii^—Co3—Co3^xii^	60.0
Co3^i^—Co0—Co2^i^	67.70 (14)	P5^xi^—Co3—Co0^viii^	159.3 (3)
Co2^ii^—Co0—Co2^i^	120.6 (2)	P5^xii^—Co3—Co0^viii^	89.59 (18)
Co2—Co0—Co2^i^	71.4 (2)	P4^viii^—Co3—Co0^viii^	53.20 (19)
Co2^iii^—Co0—Co2^i^	79.6 (2)	P5^vii^—Co3—Co0^viii^	108.5 (3)
P6—Co0—Co1	164.3 (3)	P5—Co3—Co0^viii^	52.4 (2)
P5^i^—Co0—Co1	98.8 (3)	Co3^xiii^—Co3—Co0^viii^	105.1 (2)
P4—Co0—Co1	56.2 (2)	Co3^xii^—Co3—Co0^viii^	138.58 (11)
P4^ii^—Co0—Co1	56.2 (2)	P5^xi^—Co3—Co0^ix^	89.59 (18)
Co3^iii^—Co0—Co1	62.53 (14)	P5^xii^—Co3—Co0^ix^	159.3 (3)
Co3^i^—Co0—Co1	62.53 (14)	P4^viii^—Co3—Co0^ix^	53.20 (19)
Co2^ii^—Co0—Co1	108.4 (2)	P5^vii^—Co3—Co0^ix^	52.4 (2)
Co2—Co0—Co1	108.4 (2)	P5—Co3—Co0^ix^	108.5 (3)
Co2^iii^—Co0—Co1	129.67 (15)	Co3^xiii^—Co3—Co0^ix^	105.1 (2)
Co2^i^—Co0—Co1	129.67 (15)	Co3^xii^—Co3—Co0^ix^	138.58 (11)
P6—Co0—Co1^iv^	104.29 (19)	Co0^viii^—Co3—Co0^ix^	80.8 (2)
P5^i^—Co0—Co1^iv^	158.8 (3)	P5^xi^—Co3—Co1^viii^	106.4 (3)
P4—Co0—Co1^iv^	55.3 (2)	P5^xii^—Co3—Co1^viii^	52.2 (2)
P4^ii^—Co0—Co1^iv^	55.3 (2)	P4^viii^—Co3—Co1^viii^	54.34 (19)
Co3^iii^—Co0—Co1^iv^	107.12 (19)	P5^vii^—Co3—Co1^viii^	159.6 (3)
Co3^i^—Co0—Co1^iv^	107.12 (19)	P5—Co3—Co1^viii^	93.3 (2)
Co2^ii^—Co0—Co1^iv^	63.65 (16)	Co3^xiii^—Co3—Co1^viii^	140.83 (9)
Co2—Co0—Co1^iv^	63.65 (16)	Co3^xii^—Co3—Co1^viii^	105.3 (2)
Co2^iii^—Co0—Co1^iv^	133.59 (16)	Co0^viii^—Co3—Co1^viii^	60.09 (15)
Co2^i^—Co0—Co1^iv^	133.59 (16)	Co0^ix^—Co3—Co1^viii^	107.4 (2)
Co1—Co0—Co1^iv^	60.0 (2)	P5^xi^—Co3—Co1^ix^	52.2 (2)
P5^v^—Co1—P4^v^	108.1 (2)	P5^xii^—Co3—Co1^ix^	106.4 (3)
P5^v^—Co1—P4^vi^	108.1 (2)	P4^viii^—Co3—Co1^ix^	54.34 (19)
P4^v^—Co1—P4^vi^	93.1 (4)	P5^vii^—Co3—Co1^ix^	93.3 (2)
P5^v^—Co1—P4^ii^	108.0 (3)	P5—Co3—Co1^ix^	159.6 (3)
P4^v^—Co1—P4^ii^	144.0 (3)	Co3^xiii^—Co3—Co1^ix^	140.83 (9)
P4^vi^—Co1—P4^ii^	76.3 (3)	Co3^xii^—Co3—Co1^ix^	105.3 (2)
P5^v^—Co1—P4	108.0 (3)	Co0^viii^—Co3—Co1^ix^	107.4 (2)
P4^v^—Co1—P4	76.3 (3)	Co0^ix^—Co3—Co1^ix^	60.09 (15)
P4^vi^—Co1—P4	144.0 (3)	Co1^viii^—Co3—Co1^ix^	75.95 (16)
P4^ii^—Co1—P4	92.1 (4)	P5^xi^—Co3—Co2	131.18 (19)
P5^v^—Co1—Co0	89.0 (3)	P5^xii^—Co3—Co2	131.18 (19)
P4^v^—Co1—Co0	128.2 (2)	P4^viii^—Co3—Co2	82.0 (3)
P4^vi^—Co1—Co0	128.2 (2)	P5^vii^—Co3—Co2	52.8 (2)
P4^ii^—Co1—Co0	51.9 (2)	P5—Co3—Co2	52.8 (2)
P4—Co1—Co0	51.9 (2)	Co3^xiii^—Co3—Co2	66.1 (3)
P5^v^—Co1—Co1^v^	150.6 (4)	Co3^xii^—Co3—Co2	126.1 (3)
P4^v^—Co1—Co1^v^	55.1 (2)	Co0^viii^—Co3—Co2	56.70 (15)
P4^vi^—Co1—Co1^v^	55.1 (2)	Co0^ix^—Co3—Co2	56.70 (15)
P4^ii^—Co1—Co1^v^	92.1 (2)	Co1^viii^—Co3—Co2	116.37 (18)
P4—Co1—Co1^v^	92.1 (2)	Co1^ix^—Co3—Co2	116.37 (18)
Co0—Co1—Co1^v^	120.4 (2)	Co0—P4—Co0^vii^	98.9 (4)
P5^v^—Co1—Co1^iv^	149.4 (4)	Co0—P4—Co2	70.2 (2)
P4^v^—Co1—Co1^iv^	92.6 (2)	Co0^vii^—P4—Co2	70.2 (2)
P4^vi^—Co1—Co1^iv^	92.6 (2)	Co0—P4—Co1^x^	144.1 (4)
P4^ii^—Co1—Co1^iv^	54.4 (2)	Co0^vii^—P4—Co1^x^	72.93 (17)
P4—Co1—Co1^iv^	54.4 (2)	Co2—P4—Co1^x^	74.3 (3)
Co0—Co1—Co1^iv^	60.4 (2)	Co0—P4—Co1^iv^	72.93 (17)
Co1^v^—Co1—Co1^iv^	60.0	Co0^vii^—P4—Co1^iv^	144.1 (4)
P5^v^—Co1—Co0^v^	91.0 (3)	Co2—P4—Co1^iv^	74.3 (3)
P4^v^—Co1—Co0^v^	51.8 (2)	Co1^x^—P4—Co1^iv^	93.1 (4)
P4^vi^—Co1—Co0^v^	51.8 (2)	Co0—P4—Co3^i^	69.9 (3)
P4^ii^—Co1—Co0^v^	128.0 (2)	Co0^vii^—P4—Co3^i^	69.9 (3)
P4—Co1—Co0^v^	128.0 (2)	Co2—P4—Co3^i^	116.5 (4)
Co0—Co1—Co0^v^	180.0 (2)	Co1^x^—P4—Co3^i^	133.35 (18)
Co1^v^—Co1—Co0^v^	59.6 (2)	Co1^iv^—P4—Co3^i^	133.35 (18)
Co1^iv^—Co1—Co0^v^	119.6 (2)	Co0—P4—Co1	71.84 (17)
P5^v^—Co1—Co3^iii^	54.26 (19)	Co0^vii^—P4—Co1	141.5 (4)
P4^v^—Co1—Co3^iii^	162.3 (3)	Co2—P4—Co1	133.94 (18)
P4^vi^—Co1—Co3^iii^	93.40 (17)	Co1^x^—P4—Co1	135.3 (4)
P4^ii^—Co1—Co3^iii^	53.7 (2)	Co1^iv^—P4—Co1	70.4 (2)
P4—Co1—Co3^iii^	107.1 (3)	Co3^i^—P4—Co1	72.0 (3)
Co0—Co1—Co3^iii^	57.38 (16)	Co0—P4—Co1^vii^	141.5 (4)
Co1^v^—Co1—Co3^iii^	140.25 (9)	Co0^vii^—P4—Co1^vii^	71.84 (17)
Co1^iv^—Co1—Co3^iii^	103.5 (2)	Co2—P4—Co1^vii^	133.94 (18)
Co0^v^—Co1—Co3^iii^	122.64 (18)	Co1^x^—P4—Co1^vii^	70.4 (2)
P5^v^—Co1—Co3^i^	54.26 (19)	Co1^iv^—P4—Co1^vii^	135.3 (4)
P4^v^—Co1—Co3^i^	93.40 (17)	Co3^i^—P4—Co1^vii^	72.0 (3)
P4^vi^—Co1—Co3^i^	162.3 (3)	Co1—P4—Co1^vii^	92.1 (4)
P4^ii^—Co1—Co3^i^	107.1 (3)	Co0^viii^—P5—Co1^iv^	127.8 (5)
P4—Co1—Co3^i^	53.7 (2)	Co0^viii^—P5—Co3^xiii^	131.9 (2)
Co0—Co1—Co3^i^	57.38 (15)	Co1^iv^—P5—Co3^xiii^	73.6 (3)
Co1^v^—Co1—Co3^i^	140.25 (9)	Co0^viii^—P5—Co3^xiv^	131.9 (2)
Co1^iv^—Co1—Co3^i^	103.5 (2)	Co1^iv^—P5—Co3^xiv^	73.6 (3)
Co0^v^—Co1—Co3^i^	122.64 (18)	Co3^xiii^—P5—Co3^xiv^	93.3 (4)
Co3^iii^—Co1—Co3^i^	75.95 (16)	Co0^viii^—P5—Co3	69.5 (3)
P5^v^—Co1—Co2^v^	55.41 (19)	Co1^iv^—P5—Co3	133.9 (2)
P4^v^—Co1—Co2^v^	52.7 (2)	Co3^xiii^—P5—Co3	67.5 (2)
P4^vi^—Co1—Co2^v^	105.6 (3)	Co3^xiv^—P5—Co3	131.1 (4)
P4^ii^—Co1—Co2^v^	163.3 (3)	Co0^viii^—P5—Co3^ii^	69.5 (3)
P4—Co1—Co2^v^	95.17 (19)	Co1^iv^—P5—Co3^ii^	133.9 (2)
Co0—Co1—Co2^v^	123.5 (2)	Co3^xiii^—P5—Co3^ii^	131.1 (4)
Co1^v^—Co1—Co2^v^	102.6 (2)	Co3^xiv^—P5—Co3^ii^	67.5 (2)
Co1^iv^—Co1—Co2^v^	140.73 (11)	Co3—P5—Co3^ii^	91.3 (4)
Co0^v^—Co1—Co2^v^	56.54 (14)	Co0^viii^—P5—Co2	69.7 (3)
Co3^iii^—Co1—Co2^v^	109.6 (2)	Co1^iv^—P5—Co2	73.8 (2)
Co3^i^—Co1—Co2^v^	66.10 (15)	Co3^xiii^—P5—Co2	79.62 (16)
P4—Co2—P5^vii^	103.7 (3)	Co3^xiv^—P5—Co2	147.3 (4)
P4—Co2—P5	103.7 (3)	Co3—P5—Co2	75.6 (2)
P5^vii^—Co2—P5	89.3 (3)	Co3^ii^—P5—Co2	139.2 (4)
P4—Co2—P6	103.6 (2)	Co0^viii^—P5—Co2^ii^	69.7 (3)
P5^vii^—Co2—P6	152.7 (3)	Co1^iv^—P5—Co2^ii^	73.8 (2)
P5—Co2—P6	85.31 (18)	Co3^xiii^—P5—Co2^ii^	147.3 (4)
P4—Co2—P6^vii^	103.6 (2)	Co3^xiv^—P5—Co2^ii^	79.62 (16)
P5^vii^—Co2—P6^vii^	85.30 (18)	Co3—P5—Co2^ii^	139.2 (4)
P5—Co2—P6^vii^	152.7 (3)	Co3^ii^—P5—Co2^ii^	75.6 (2)
P6—Co2—P6^vii^	87.33 (17)	Co2—P5—Co2^ii^	89.3 (3)
P4—Co2—Co0^vii^	53.20 (19)	Co0^i^—P6—Co0^viii^	120.0
P5^vii^—Co2—Co0^vii^	90.21 (18)	Co0^i^—P6—Co0	120.0
P5—Co2—Co0^vii^	155.9 (3)	Co0^viii^—P6—Co0	120.0
P6—Co2—Co0^vii^	105.4 (2)	Co0^i^—P6—Co2^xv^	69.23 (17)
P6^vii^—Co2—Co0^vii^	51.06 (12)	Co0^viii^—P6—Co2^xv^	68.36 (17)
P4—Co2—Co0	53.20 (19)	Co0—P6—Co2^xv^	136.33 (9)
P5^vii^—Co2—Co0	155.9 (3)	Co0^i^—P6—Co2^iii^	68.36 (17)
P5—Co2—Co0	90.21 (18)	Co0^viii^—P6—Co2^iii^	136.33 (9)
P6—Co2—Co0	51.06 (12)	Co0—P6—Co2^iii^	69.23 (17)
P6^vii^—Co2—Co0	105.4 (2)	Co2^xv^—P6—Co2^iii^	77.58 (13)
Co0^vii^—Co2—Co0	80.6 (2)	Co0^i^—P6—Co2^i^	68.36 (17)
P4—Co2—Co0^viii^	140.20 (12)	Co0^viii^—P6—Co2^i^	136.33 (9)
P5^vii^—Co2—Co0^viii^	106.2 (3)	Co0—P6—Co2^i^	69.23 (17)
P5—Co2—Co0^viii^	51.6 (2)	Co2^xv^—P6—Co2^i^	137.59 (6)
P6—Co2—Co0^viii^	50.70 (12)	Co2^iii^—P6—Co2^i^	87.34 (17)
P6^vii^—Co2—Co0^viii^	104.5 (2)	Co0^i^—P6—Co2^viii^	69.23 (17)
Co0^vii^—Co2—Co0^viii^	150.1 (2)	Co0^viii^—P6—Co2^viii^	68.36 (17)
Co0—Co2—Co0^viii^	92.22 (16)	Co0—P6—Co2^viii^	136.33 (9)
P4—Co2—Co0^ix^	140.20 (12)	Co2^xv^—P6—Co2^viii^	87.34 (17)
P5^vii^—Co2—Co0^ix^	51.6 (2)	Co2^iii^—P6—Co2^viii^	137.59 (6)
P5—Co2—Co0^ix^	106.2 (3)	Co2^i^—P6—Co2^viii^	77.58 (13)
P6—Co2—Co0^ix^	104.5 (2)	Co0^i^—P6—Co2^ii^	136.33 (9)
P6^vii^—Co2—Co0^ix^	50.70 (12)	Co0^viii^—P6—Co2^ii^	69.23 (17)
Co0^vii^—Co2—Co0^ix^	92.22 (16)	Co0—P6—Co2^ii^	68.37 (17)
Co0—Co2—Co0^ix^	150.1 (2)	Co2^xv^—P6—Co2^ii^	77.58 (13)
Co0^viii^—Co2—Co0^ix^	79.6 (2)	Co2^iii^—P6—Co2^ii^	77.58 (13)
P4—Co2—Co1^x^	52.99 (19)	Co2^i^—P6—Co2^ii^	137.59 (6)
P5^vii^—Co2—Co1^x^	50.7 (2)	Co2^viii^—P6—Co2^ii^	137.59 (6)
P5—Co2—Co1^x^	102.3 (3)	Co0^i^—P6—Co2	136.33 (9)
P6—Co2—Co1^x^	156.4 (2)	Co0^viii^—P6—Co2	69.23 (17)
P6^vii^—Co2—Co1^x^	94.84 (11)	Co0—P6—Co2	68.37 (17)
Co0^vii^—Co2—Co1^x^	59.81 (15)	Co2^xv^—P6—Co2	137.59 (6)
Co0—Co2—Co1^x^	106.0 (2)	Co2^iii^—P6—Co2	137.59 (6)
Co0^viii^—Co2—Co1^x^	148.9 (2)	Co2^i^—P6—Co2	77.58 (13)
Co0^ix^—Co2—Co1^x^	94.89 (12)	Co2^viii^—P6—Co2	77.58 (13)
P4—Co2—Co1^iv^	52.99 (19)	Co2^ii^—P6—Co2	87.33 (17)

Symmetry codes: (i) −*y*, *x*−*y*, *z*; (ii) *x*, *y*, *z*−1; (iii) −*y*, *x*−*y*, *z*−1; (iv) −*y*+1, *x*−*y*+1, *z*; (v) −*x*+*y*, −*x*+1, *z*; (vi) −*x*+*y*, −*x*+1, *z*−1; (vii) *x*, *y*, *z*+1; (viii) −*x*+*y*, −*x*, *z*; (ix) −*x*+*y*, −*x*, *z*+1; (x) −*y*+1, *x*−*y*+1, *z*+1; (xi) −*y*+1, *x*−*y*, *z*+1; (xii) −*y*+1, *x*−*y*, *z*; (xiii) −*x*+*y*+1, −*x*+1, *z*; (xiv) −*x*+*y*+1, −*x*+1, *z*−1; (xv) −*x*+*y*, −*x*, *z*−1.
